# Activation of Brainstem Neurons During Mesencephalic Locomotor Region-Evoked Locomotion in the Cat

**DOI:** 10.3389/fnsys.2019.00069

**Published:** 2019-11-14

**Authors:** Ioan Opris, Xiaohong Dai, Dawn M. G. Johnson, Francisco J. Sanchez, Luz M. Villamil, Songtao Xie, Cecelia R. Lee-Hauser, Stephano Chang, Larry M. Jordan, Brian R. Noga

**Affiliations:** ^1^The Miami Project to Cure Paralysis, Department of Neurological Surgery, University of Miami Miller School of Medicine, Miami, FL, United States; ^2^Department of Physiology, Spinal Cord Research Centre, University of Manitoba, Winnipeg, MB, Canada

**Keywords:** mesencephalic locomotor region, cuneiform nucleus, pedunculopontine nucleus, fictive locomotion, reticulospinal, monoamine, choline acetyltransferase, activity-dependent labeling

## Abstract

The distribution of locomotor-activated neurons in the brainstem of the cat was studied by c-*Fos* immunohistochemistry in combination with antibody-based cellular phenotyping following electrical stimulation of the mesencephalic locomotor region (MLR) – the anatomical constituents of which remain debated today, primarily between the cuneiform (CnF) and the pedunculopontine tegmental nuclei (PPT). Effective MLR sites were co-extensive with the CnF nucleus. Animals subject to the locomotor task showed abundant *Fos* labeling in the CnF, parabrachial nuclei of the subcuneiform region, periaqueductal gray, locus ceruleus (LC)/subceruleus (SubC), Kölliker–Fuse, magnocellular and lateral tegmental fields, raphe, and the parapyramidal region. Labeled neurons were more abundant on the side of stimulation. In some animals, *Fos*-labeled cells were also observed in the ventral tegmental area, medial and intermediate vestibular nuclei, dorsal motor nucleus of the vagus, n. tractus solitarii, and retrofacial nucleus in the ventrolateral medulla. Many neurons in the reticular formation were innervated by serotonergic fibers. Numerous locomotor-activated neurons in the parabrachial nuclei and LC/SubC/Kölliker–Fuse were noradrenergic. Few cholinergic neurons within the PPT stained for *Fos*. In the medulla, serotonergic neurons within the parapyramidal region and the nucleus raphe magnus were positive for *Fos*. Control animals, not subject to locomotion, showed few *Fos*-labeled neurons in these areas. The current study provides positive evidence for a role for the CnF in the initiation of locomotion while providing little evidence for the participation of the PPT. The results also show that MLR-evoked locomotion involves the parallel activation of reticular and monoaminergic neurons in the pons/medulla, and provides the anatomical and functional basis for spinal monoamine release during evoked locomotion. Lastly, the results indicate that vestibular, cardiovascular, and respiratory centers are centrally activated during MLR-evoked locomotion. Altogether, the results show a complex pattern of neuromodulatory influences of brainstem neurons by electrical activation of the MLR.

## Introduction

Of the various higher brain centers that elicit locomotion when stimulated, the MLR, a key, phylogenetically preserved, regulatory node within the supraspinal locomotor circuit controlling spinal locomotor neurons (Shik et al., 1966, 1967; Grillner et al., 2008; Jordan et al., 2008), is increasingly looked at as a target for improving locomotion (freezing-of-gait) in Parkinson’s disease (PD) and after spinal cord injury (SCI). The anatomical equivalent of this physiologically defined region was originally thought to be the CnF (Shik et al., 1966) and subsequent work confirmed this conclusion (Mori et al., 1989, 1992; Grillner et al., 1997; Jordan, 1998; Jordan and Sławińska, 2014; Takakusaki et al., 2016). The nearby cholinergic PPT has also been suggested to be the primary component of the MLR (Garcia-Rill et al., 1986, 1987, 2011). Recent optogenetic and chemogenetic experiments, however, have cast doubt on the role of the cholinergic PPT neurons in the initiation of locomotion (Lee et al., 2014; Roseberry et al., 2016; Capelli et al., 2017; Kroeger et al., 2017; Caggiano et al., 2018; Josset et al., 2018). Rather, these studies emphasize the key role for glutamatergic neurons, especially in CnF and SubCnF regions, for initiating locomotion and suggest that cholinergic neurons may only have a role in the modulation of ongoing locomotor activity or play a role in non-locomotor functions of the MLR.

The MLR does not directly project to the spinal cord but rather activates spinal neurons controlling locomotion (Noga et al., 1995, 2003; Dai et al., 2005) by activation of reticulospinal (RS) neurons in the brainstem (Shik et al., 1967; Orlovskii, 1970; Shefchyk et al., 1984; Garcia-Rill and Skinner, 1987; Noga et al., 1988, 1991, 2003). These in turn descend through the ventral funiculus (Steeves and Jordan, 1984; Noga et al., 1991, 2003). This pathway, considered to be the “command pathway” for the initiation of locomotion (Shik et al., 1967; Jordan, 1998), activates spinal locomotor neurons, in part, by the release of glutamate (Douglas et al., 1993; Hägglund et al., 2010). Such results are supported by optogenetic studies in the mouse, which were used to stimulate glutamatergic RS neurons within the lateral paragigantocellular (LPGi) nucleus (Capelli et al., 2017). Photo-stimulation of these neurons evokes short-latency high-speed locomotion, while ablation of this population significantly reduces the speed of glutamatergic MLR-evoked locomotion. LPGi neurons receive a predominant glutamatergic input from the CnF (Capelli et al., 2017). Glutamatergic RS neurons expressing the transcription factors Lhx3 and/or Chx10 within the MedRF that are activated during locomotion and receive anatomical inputs from the MLR have also been described (Bretzner and Brownstone, 2013), supporting this concept.

In addition to RS command neurons, there is evidence that monoaminergic neurons may play a key role in the activation of spinal locomotor networks. For example, intravenous administration of noradrenergic and serotonergic precursors produces reflex discharges that resemble locomotion (Jankowska et al., 1967; Viala and Buser, 1969). Since then, many studies have shown that monoaminergic drugs may evoke or modulate locomotion in spinally injured cats (Barbeau and Rossignol, 1991; Kiehn et al., 1992; Marcoux and Rossignol, 2000), rats (Cazalets et al., 1992; Kiehn and Kjærulff, 1996; Feraboli-Lohnherr et al., 1999; Sqalli-Houssaini and Cazalets, 2000; Antri et al., 2002), and mice (Christie and Whelan, 2005). Since MLR stimulation produces a similar effect as seen with L-DOPA administration to the spinal cord, it was suggested that the MLR activates a noradrenergic descending system which controls the spinal locomotor generating network (Grillner and Shik, 1973). This idea is supported by the presence of catecholamine-containing neurons in the vicinity of the MLR (Steeves et al., 1976), the demonstration of direct projections from the MLR to the monoaminergic nuclei (Edwards, 1975; Steeves and Jordan, 1984; Sotnichenko, 1985) and the observation that both noradrenergic (Rasmussen et al., 1986) and serotonergic neurons are rhythmically active during overground or treadmill locomotion (Veasey et al., 1995). Recent work in our laboratory has now shown that during MLR-evoked locomotion, spinal monoamine release is widespread and modulated on a timescale of seconds, in tandem with centrally generated locomotion (Noga et al., 2017). While this release is observed during MLR-evoked locomotion, it is not obligatory since depletion of spinal NE or 5-HT does not abolish the MLR’s ability to evoke locomotion (Steeves et al., 1980).

To enable MLR-evoked locomotion the activity within brainstem microcircuits must be modulated. In this study we aimed to identify the brainstem neurons activated by electrical stimulation of the MLR as this method is the current clinical standard for targeted stimulation of deep brain structures. MLR sites were identified by their low electrical thresholds and best locomotor responses to stimulation. In the first series of experiments, we documented the distribution of locomotor-activated neurons within the mesencephalon, pons, and medulla using c-*Fos* immunohistochemistry (IHC) (Herdegen and Leah, 1998) as an activity-dependent marker of induced locomotion (Huang et al., 2000; Dai et al., 2005; Noga et al., 2009, 2011). To gain perspective on cells potentially generating locomotor movements, i.e., those that are centrally activated in the absence of peripheral afferent feedback, we used the fictive locomotion preparation in which animals are paralyzed by neuromuscular blockade and locomotor activity is monitored by electroneurogram (ENG) recordings from peripheral nerves. Animals subject to treadmill locomotion, with consequent phasic, sensory feedback were also examined for comparative purposes. In a second series of experiments, *Fos*+ cells were inspected for co-localization with either dopamine-beta-hydroxylase (DβH), 5-hydroxytryptamine (5-HT), or choline acetyltransferase (ChAT) to determine whether noradrenergic, serotonergic, or cholinergic neurons are activated during MLR-evoked fictive locomotion. The results reveal the anatomical correlate of the MLR, the target descending locomotor pathway neurons and provide evidence for a central coupling of locomotor, vestibular, respiratory, and cardiovascular networks during locomotion. Preliminary results have been reported (Noga et al., 2008).

## Materials and Methods

### Animal Preparation

Experimental procedures were approved by the local University IACUC committees in accordance with the National Institute of Health guidelines (NIH Publications No. 80-23; revised 1996). The number of animals used, and their pain and distress, were minimized. Experiments were performed on 10 adult female cats weighing between 1.9 and 4.3 kg subject to precollicular–postmammillary decerebration. Experimental procedures for treadmill and fictive locomotion experiments were as described previously (Dai et al., 2005; Noga et al., 2009). For fictive locomotion experiments, nerves to one flexor and extensor muscle supplying each of the hindlimbs and forelimbs were dissected, bilaterally, and mounted in tunnel electrodes. The head of each animal was fixed in a Transvertex headframe. In treadmill-locomotion (TL-1) and treadmill-control (TC-1 and TC-2) experiments, all four limbs were free to step on a treadmill belt, and the hindquarters were suspended by a sling under the abdomen. In fictive-locomotion (FL-1,2,3) and fictive-control (FC-1,2,3,4) experiments, the animals were suspended with all four limbs pendant. Table 1 summarizes the procedural details of animals included in the present study.

**TABLE 1:** Animals and experimental procedures.

**ID**	**Study**	**Total time locomotion (min)**	**Time end of stim to perfusion (h)**	**Time decerebration to perfusion (h)**	**IHC procedures**	**Previous study designation**
FL-1	1	157	1	9	DAB (Fos)	FL-1^†^
FL-2	1	210	1	9	DAB (Fos)	FL-2^†^
FL-3	2	262	1	10	Fluorescence (Fos, DβH, ChAT, 5-HT)	LC-1^††^
FC-1	1	<1	6	8.5	DAB (Fos)	FC-1^†^
FC-2	1	<1	6	8.5	DAB (Fos)	FC-2^†^
FC-3	1	No stim	–	9	DAB (Fos)	FC-4^†^
FC-4	2	<1	8	10	Fluorescence (Fos, DβH, ChAT, 5-HT)	C^††^
TL-1	1	322	1	9	DAB (Fos)	TL-1^†^
TC-1	1	<1	7	8.5	DAB (Fos)	TC-1^†^
TC-2	1	No stim	–	9	DAB (Fos)	TC-2^†^

*Animals are assigned into different groups: fictive locomotion (FL); treadmill locomotion (TL); fictive control (FC); and treadmill control (TL). ^†^Dai et al. (2005) and ^††^Noga et al. (2009, 2011).*

### Stimulation and Recording

**FIGURE 1:**
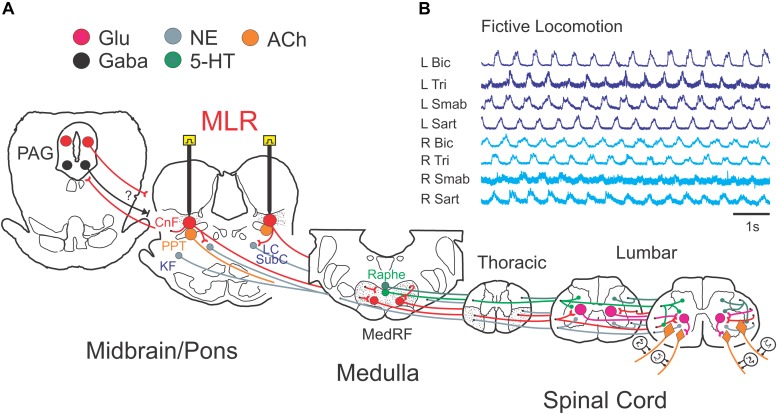
Experimental schema illustrating the brainstem network involved in neuromodulation of locomotor command with stimulation of the MLR. **(A)** Schematic diagram of the brainstem showing the putative anatomical correlates of the MLR, the cuneiform (CnF), and pedunculopontine tegmental (PPT) nuclei, and their putative target neurons within the midbrain/pons and medulla: the periaqueductal gray (PAG), the glutamatergic reticulospinal (RS) neurons within the medial reticular formation (MedRF), the noradrenergic nuclei locus ceruleus (LC), subceruleus (SubC) and Kölliker–Fuse (KF), and the serotonergic nuclei (Edwards, 1975; Steeves and Jordan, 1984; Sotnichenko, 1985). Axons of RS, noradrenergic and serotonergic neurons innervate locomotor central pattern generator neurons (Steeves and Jordan, 1980; Noga et al., 2003, 2009, 2011, 2017). The projections of neurons releasing the neurotransmitters glutamate (Glu), acetylcholine (ACh), noradrenaline (NE), serotonin (5-HT), and gamma-aminobutyric acid (GABA) are depicted across the major relays in locomotion control. **(B)** Example of fictive locomotor activity as monitored by electroneurogram (ENG) recordings from peripheral nerves from all limbs. R, right; L, left; Bic, biceps brachii; Tri, triceps brachii; Smab, semimembranosus/anterior biceps, Sart, sartorius.

**FIGURE 2:**
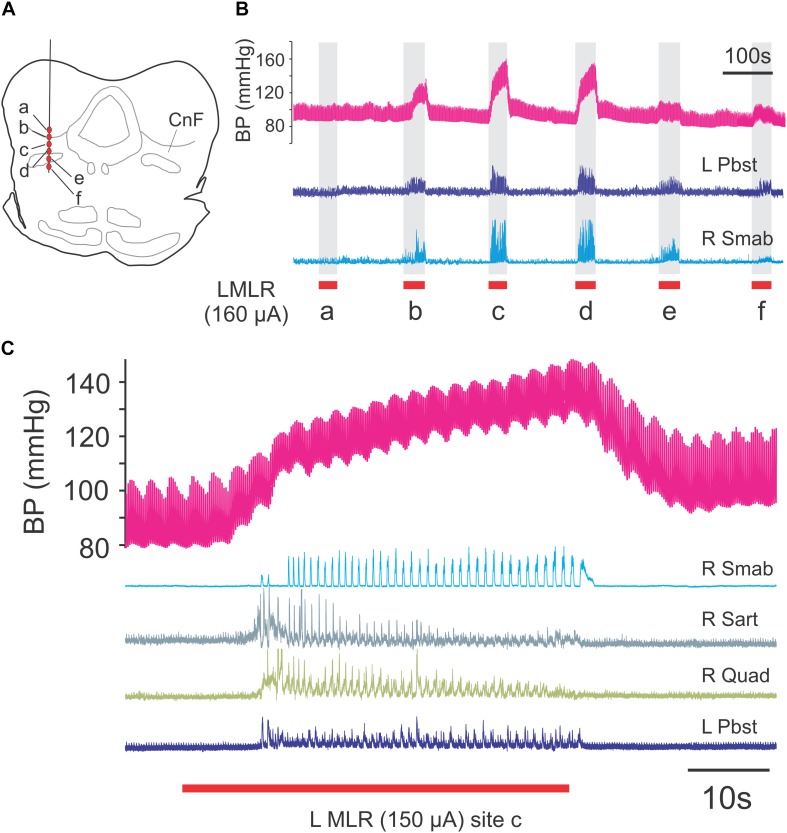
Mapping of locomotor responses to electrical stimulation of the MLR and surrounding region of the midbrain. Stimulation of CnF and SubCnF sites **(A)** produces largest locomotor and pressor responses **(B)** in neuromuscular paralyzed and artificially ventilated decerebrate cats. Note reduced ENG responses in ventral stimulation locations. Locomotion monitored by electroneurogram recordings from hindlimb peripheral nerves. Blood pressure responses measured from indwelling catheter (carotid artery). **(C)** Locomotor and pressure responses to stimulation of site *c*. Low amplitude pressor responses (∼0.4 Hz) result from artificial respiration of the animal. R, right; L, left; Pbst: posterior biceps/semitendinosus; Smab, semimembranosus/anterior biceps; Sart, sartorius; Quad, quadriceps.

The experimental setup is illustrated in Figure 1. Following a recovery period from the decerebration of 1.5–3 h, 4-limbed locomotion was evoked by electrical stimulation of the MLR (1.0 ms square wave pulses, 15–20 Hz) using monopolar stimulating electrodes (SNE-300; David Kopf Instruments, Tujunga, CA, United States) as previously described (Noga et al., 2009, 2011). Electrodes were stereotaxically inserted into the mesopontine tegmentum at an area bounded by posterior (P) 1–3 and lateral (L) 3.0–5.0 mm and included the CnF, bcm within the SubCnF region, and the PPT. Electrodes were typically advanced slowly while stimulating, thus limiting the stimulation of unrelated sites, until the optimal locomotor response was obtained. Thresholds were then tested. If no response was noted or if stimulation strength was high, the electrode was repositioned, and the procedure repeated. Final position was determined by the best locomotor response (greatest ENG amplitudes presenting in locomotor-like rhythms) provided by the lowest threshold at the specified frequency and pulse width. Tract coordinates and electrode depth were noted. In some experiments, electrodes were repositioned in small incremental steps and responses to electrical stimulation at the same strength (slightly above predetermined thresholds) were examined (Figure 2) as a further validation of the threshold test results. During the experiment, the strength of stimulation was adjusted to a level which was suitable to maintain locomotion for prolonged periods. Locomotion was monitored by visual confirmation of weight support and walking on the treadmill (treadmill experiments: belt speed: ∼0.46 m/s) or from ENG recordings (fictive locomotion experiments). Representative ENG activity was obtained from the bouts of locomotion throughout the stimulation period (Figures 1B, 2). The ENG signals were amplified with AC-coupled amplifiers (bandwidth 300 Hz to 10 kHz), rectified and low-pass filtered (10 or 20 ms time constant), and subsequently digitized through a 1 MHz, 16 channel analog-to-digital converter (12 bit) at 2–4 kHz using customized software (Spinal Cord Research Centre, University of Manitoba, Canada).

### Tissue Perfusion

In all of the experiments reported here, there was an 8.5–10 h interval between decerebration and perfusion to reduce *Fos* expression resulting from surgical procedures (Table 1). At the end of each locomotor experiment, after a 1 h interval with no-stimulation and immediately prior to perfusion, a small electrolytic lesion was made to mark the MLR stimulation site(s). Animals were re-anesthetized with either halothane or sodium pentobarbital (30 mg/kg) and perfused transcardially with normal saline (0.3 ml/g of animal weight) containing 0.1% NaNO_2_ and 100 units/ml heparin, followed by 4% paraformaldehyde, 0.2% picric acid, in 0.1 M phosphate-buffered saline (PBS, 4°C), pH 7.4 (1 ml/g of animal weight). The brainstems were removed, post-fixed in the fixative solution for 5 h, and cryoprotected by washing in a solution containing 25% sucrose, 10% glycerol, and 0.001% sodium azide in 0.1 M phosphate buffer for several days.

### Immunohistochemistry

The immunohistochemical analysis was carried out on brainstem tissue obtained from animals described in our previous publications on MLR-evoked spinal cord *Fos* expression (Dai et al., 2005; Noga et al., 2009, 2011). Table 1 summarizes the designations (animal ID) for the present study and from previous studies. Frozen tissue sections of 20 (Dai et al., 2005) or 30 μm (Noga et al., 2009, 2011) thickness were sectioned in a sagittal or coronal plane with a sliding microtome and collected in 0.1 M PBS. To optimize immunohistochemical procedures, a small group of sections were randomly collected from the brainstem segments and a primary antibody dilution series performed. In addition, for the pre-adsorption control, cat tissue sections were incubated only with pre-immuno serum without the primary antibodies. Immunoreactivity was totally absent after omission of all primary antibodies. Controls conducted for double labeling demonstrated no cross-reactivity between primary antibodies and inappropriate secondary antibodies. Selected serial sections of the brainstem were processed to label c-*Fos* nuclear protein alone or co-localized with either DβH, 5-HT, or ChAT to identify activated noradrenergic, serotonergic, or cholinergic brainstem neurons. Two experimental protocols were followed. In Study 1, examining the distribution of activated neurons, *Fos* was stained using diaminobenzidine (DAB) IHC (Dai et al., 2005). Sections were incubated for 72 h in sheep polyclonal anti-*Fos* IgG (Cambridge Research Biochemical) 1:2,000. In Study 2, we examined the distribution of *Fos*-activated noradrenergic, serotonergic, or cholinergic neurons. Cells were stained for *Fos*, and either DβH, 5-HT, or ChAT using fluorescent immunohistochemical techniques (Noga et al., 2009, 2011). Sections were incubated 48 h in rabbit polyclonal anti-*Fos* IgG (PC38-100U: Oncogene Research Products/Calbiochem, San Diego, CA, United States) 1:2,500. Sections were then incubated for 48 h in either mouse monoclonal anti-DβH IgG (MAB308: Chemicon International, Temecula, CA, United States) 1:500, rat monoclonal anti-5-HT IgG (MAB352: Chemicon International, Temecula, CA, United States) 1:100, or goat polyclonal anti-ChAT IgG (AB144P: Millipore) 1:100. Each secondary antibody was conjugated to a different fluorophore (Molecular Probes-Invitrogen, Carlsbad, CA, United States): Alexa 488 (green) for *Fos* (1:500; goat anti-rabbit, A-11008), Alexa 594 (red) for DβH (1:500; goat anti-mouse, A-11005), Alexa 594 for 5-HT (1:200; goat anti-rat; A-11007), and Alexa 594 for ChAT (1:200; donkey anti-goat).

### Data Analysis and Interpretation

Anatomical landmarks from sagittal or coronal brainstem sections were identified using an atlas of the cat brainstem (Berman, 1968). The location of the stimulation sites were determined from depth measurements taken from the surface of the IC of the electrode along the reconstructed electrode tracks and also from a small electrolytic lesions made in the MLR prior to perfusion. For DAB experiments, sections were examined under a light microscope, and cellular architecture, as well as locations of labeled cells, were drawn using a camera lucida. For co-localization experiments, sections were examined with Zeiss Axioline microscopes using fluorescence microscopy. Cells were mapped using Neurolucida software. Cell counts were done using stereologic cell counting methods (Stereo Investigator 5.0, Microbrightfield Bioscience, Inc., Williston, VT, United States) giving estimates of cell number per sections and or nuclei. Cell positions of labeled neurons were determined by reconstruction of individual images of each section at 10× power. Confocal microscopy (Zeiss LSM510, with Ar multiline and HeN1 564) was used for high power examination of the three-dimensional structure of selected cells. Noradrenergic, cholinergic, and serotonergic cells were scanned in a series of optical sections and three-dimensional reconstructions were digitized. Serotonergic innervation of *Fos*-labeled reticular neurons (Di Prisco et al., 1994; Antri et al., 2008) was assessed using criteria previously established for spinal locomotor activated neurons (Noga et al., 2009).

## Results

Locomotion was evoked by stimulation of the MLR for a period of 2.5–5.5 h in four animals (FL-1, FL-2, FL-3, and TL-1; Table 1). In three animals (FL-1, FL-2, and TL-1), MLR stimulation was confined to one side (Study 1). In FL-3, both sides were stimulated during the testing period (Study 2). Control animals received the same surgical procedures as the locomotor test animals, except that they were not subject to the full locomotor task. Most control animals (FC-1, FC-2, FC-4, and TC-1) could produce locomotion with MLR stimulation but were only briefly stimulated. Sub-optimal sites were stimulated only briefly during the search for low threshold sites (see the section “Materials and Methods”), limiting their possible contribution to overall *Fos* expression. Two animals (FC-3 and TC-2) were neither stimulated nor had stimulating electrodes inserted.

### Study 1: *Fos*-Labeled Cells in the Brainstem

**FIGURE 3:**
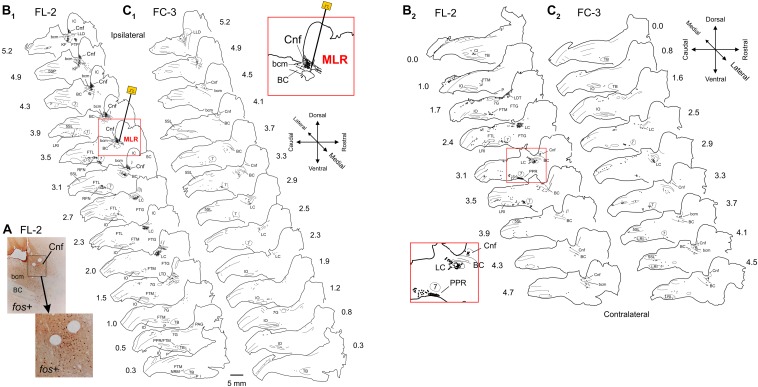
Distribution of *Fos* labeled neurons in the brainstem of a MLR-evoked fictive locomotor cat compared to non-locomotor control cat. **(A)** Photomicrographs illustrating the appearance of *Fos* labeled neurons near the stimulation electrode in the CnF and SubCnF region. Inset: higher magnification image of indicated area. **(B_1_,_2_,C_1_,_2_)** Camera lucida drawings showing distribution of *Fos* immunoreactive neurons in brainstem of fictive locomotor cat FL-2 and non-locomotor control cat FC-3, respectively. Images in **(B_1_)** and **(B_2_)** show distribution on side ipsilateral and contralateral to the stimulating electrode (indicated in sagittal section at 3.5 mm from midline), respectively. Images in **(C_1_)** and **(C_2_)** show distribution on right and left sides of the brainstem, respectively. Note dense labeling within the CnF, the bcm of the SubCnF region, the locus ceruleus (LC), the lateral tegmental field (FTL), the magnocellular tegmental field (FTM), and the parapyramidal region (PPR). Labeling was more robust on side of stimulation. Control animals show relatively low numbers of labeled cells. Each diagram includes all labeled cells from single sections at the indicated levels. Each dot represents one labeled cell in this and other figures. Insets: higher magnification of MLR stimulation site in **(B_1_)** (lateral 3.5 mm) and LC/FTL/PPR region in **(B_2_)** (contralateral 3.1 mm). Anatomical structures labeled in this and other figures are listed under Abbreviations.

Sagittal sections of the brainstems from two fictive locomotion animals (FL-1 and FL-2), one treadmill locomotion (TL-1), and five control animals (FC-1–3, TC-1, and TC-2) were stained for *Fos* using the DAB method and examined under the microscope. Representative photomicrographs illustrating the appearance of *Fos*-labeled neurons in the CnF and the SubCnF region (bcm) from FL-2 cat are shown in Figure 3A.

#### Locomotor Experiments

In animals subject to the locomotion protocol, the best locomotor responses were observed with stimulation within the CnF and SubCnF region (bcm), dorsal to the BC (Figure 2). At the frequencies used (15–20 Hz), the response produced by stimulation in more ventral sites including the BC and/or PPT was either of lower amplitude or consisted of erratic or tonic nerve activity. Stimulation of the MLR invariably increased blood pressure during the stimulation period, the amplitude of which was highest during stimulation of the best locomotor points (Figure 2). Stimulation strengths adjusted to maintain locomotor bouts over long periods of time ranged between 50 and 160 μA.

**FIGURE 4:**
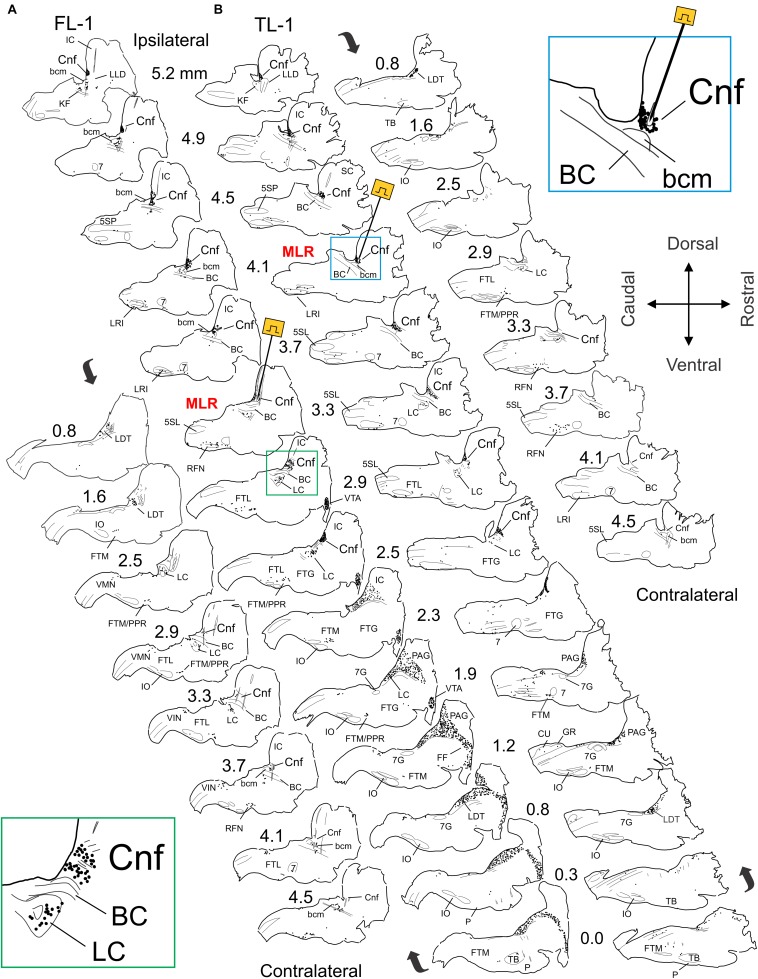
Distribution of *Fos* labeled neurons in the brainstem of MLR-evoked fictive locomotor cat 1 (FL-1) and treadmill locomotor cat 1 (TL-1). **(A,B)** Camera lucida drawings showing distribution of *Fos* immunoreactive neurons in brainstem. Site of MLR stimulation electrodes indicated in sagittal sections at 3.3 and 4.1 mm lateral to midline for FL-1 **(A)** and TL-1 **(B)**, respectively. Note dense labeling within the CnF, bcm, the laterodorsal tegmental nucleus (LDT), the LC, the FTL, the FTM, and the PPR in both animals. Strong labeling was also observed in the periaqueductal gray (PAG) and ventral tegmental area (VTA) of FL-1 and less so in the PAG of TL-1. Labeling was more robust on side of stimulation. Insets: higher magnification of CnF/LC region in FL-1 (**A:** ipsilateral 2.9 mm) and MLR stimulation site in TL-1 (**B:** lateral 3.5 mm).

The distribution of *Fos*-labeled neurons from three locomotor animals is presented in Figures 3 (FL-2) and 4 (FL-1 and TL-1). Overall, the distribution of labeled cells within fictive and treadmill locomotor animals was similar, indicating that *Fos* expression is governed more by the central drive than by afferent feedback. Labeled neurons were observed in several brainstem nuclei and were typically greater in number on the side of stimulation. (1) CnF: large numbers of cells were labeled in the CnF on the side of stimulation (*insets*, Figures 3, 4). Fewer cells were observed in the contralateral CnF (e.g., FL-1; Figure 4). (2) bcm: cells in the bcm of the SubCnF region were labeled in all animals. (3) LC: cells in the LC were labeled bilaterally in all locomotor cats (Figure 4, *inset*). (4) KF: labeled cells were found bilaterally in the area of the KF. (5) PPT: a small group of labeled cells located rostral to the bcm and KF, in lateral sections on the side of stimulation (lateral L4.3–5.2) was seen in cat FL-2 (Figure 3B_1_) but not cats FL-1 and TL-1 (Figure 4). *Fos*+ neurons were also observed more caudally in an area medial and ventral to the BC. The phenotype(s) of these neurons is not clear without ChAT immunostaining (Garcia-Rill et al., 1987) and cells in this area overlap with cells of the LC. (6) Laterodorsal tegmental nucleus (LDT): *Fos*+ cells were observed in the LDT on the ventromedial border of the caudal ventrolateral PAG in all locomotor animals (Figure 3B_1_ – L0.5–1.5 and Figures 4A,B – L0.8–1.2). (7) PAG: many *Fos*-labeled neurons of the ipsilateral PAG were labeled at-level and rostral to the site of stimulation in FL-Cat1 (Figures 3B1,2, 4A,B). A small number of cells within the PAG of TL-1 was also labeled. (8) FF: a column of *Fos*+ cells extended rostrally and ventrally from the PAG (Figure 4A – L0–1.2) through the central gray and the FF toward the VTA of FL-1. (9) The VTA of Tsai was labeled on the side of stimulation of cat FL-1 (Figures 3B1,2, 4A,B – L1.9–2.9). It was not possible to evaluate the contralateral VTA since tissue along the cut edge of the brain (decerebration) was contaminated with blood cells. (10) FTL: numerous cells were labeled bilaterally in the FTL (L4.0–2.0) in all locomotor animals (Figures 3B, 4A,B). Most cells were located in an area bounded rostrally by the facial nucleus (7N), caudally by the LRI, and ventrally by the inferior olivary nucleus (IO) (Figure 3B_2_, *inset*). Few, if any, cells were found in the LRI or the trigeminal nuclei (5SL and 5SP). Around L3, the length of this cell column shortened and was concentrated in the medulla near the ponto-medullary junction. The labeled cells in this area overlapped the areas occupied by the AMB and the RFN. (11) FTM: *Fos*-labeled cells were found bilaterally in an area immediately caudal to the TB and rostral to the IO from about L0.5 to 2.0 at the ponto-medullary junction in all locomotor animals. Some labeled cells also appeared in an area just dorsal to the TB. Cells toward the midline were in the region of the NRM and obscurus (NRO). (12) Dorsal medulla: a small group of cells located bilaterally in the dorsal medulla was labeled in locomotor cats (Figures 3B, 4A,B). Most of the cells were located in the area of the NTS, the dmnV, and the vestibular nuclei (VLN) (VIN, VMN) (L1–3.5). (13) CU and GR: a few labeled neurons were observed in the CU and GR of TL-1 (Figure 4B, L1.2) but not in FL-1 and FL-2. (14) Central canal: a strip of labeled neurons were observed surrounding the central canal in caudal brainstem sections of FL-2 (Figure 3B_1_) but was not observed in FL-1 and TL-1.

#### Non-locomotor Control Experiments

**FIGURE 5:**
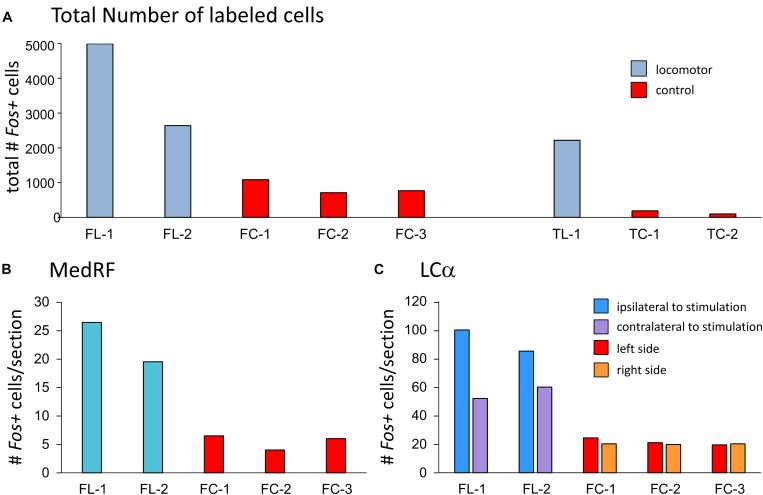
Comparisons of numbers of *Fos*-labeled cells within the brainstem, MedRF, and locus ceruleus alpha (LCα) of MLR-evoked locomotor and control cats. The total number of labeled cells in both fictive and treadmill locomotor animals exceeds that observed in respective control animals **(A)**. Likewise, the number of *Fos*+ cells/section within the MedRF **(B)** and LCα **(C)** was consistently much higher that observed in control animals. The number of *Fos*-labeled cells/section in the LCα was higher on the side of stimulation.

Limited labeling in the various brainstem nuclei was seen in control animals, including those that were stimulated only briefly to ensure that the brainstem health was comparable to locomotor animals. Many hours elapsed between this brief locomotor bout and perfusion (Table 1) to minimize *Fos* expression in the Control animals. Labeling was not observed in the CnF nucleus of FC-3 (Figures 3C1,2), the non-stimulated control, although sparse labeling was seen in the bcm. This animal was representative of the other fictive locomotion cats and showed higher numbers of labeled cells in comparison to treadmill control animals (Figure 5A). The increased numbers of cells in the fictive-control animals as compared with the treadmill-control animals is most likely due to the additional sensory input produced by the nerve dissection surgery and placement of the animals in the spinal frame.

#### Comparisons of the Number of Labeled Cells in Locomotor and Control Animals

*Fos* labeling in the brainstem, MedRF, and LC of locomotor animals was much higher than that seen in their respective controls (Figure 5). In locomotor animals, the total number of labeled cells was highest in FL-1. This was primarily due to the large number of labeled cells within the PAG, although there was a scattering of labeled cells in the PAG of TL-1 (Figure 4: 1.9 and 1.2 lateral to midline). Inspection of panels from Figures 3, 4 show that the number of labeled cells was also greater on the side of stimulation than on the opposite, unstimulated side. This is shown for the LC of locomotor animals in Figure 5C.

### Study 2: Phenotyping of *Fos*-Labeled Cells in the Brainstem

A second set of experiments were done combining *Fos* IHC with staining for either DβH, ChAT, or 5-HT to identify noradrenergic, cholinergic, and serotonergic neurons, respectively. As in Study 1 animals, the best locomotor responses were obtained with stimulation within the CnF and SubCnF region (bcm), dorsal to the BC. Four-limbed locomotion was evoked by stimulation of both sides of the brainstem, either separately or together to maintain locomotor bouts lasting upward of 100 min at a time for a total of 262 min. Stimulation in more ventral sites failed to induce coordinated locomotion and instead evoked erratic or tonic nerve activity. The brainstem viability was comparable in control (FC-4) and locomotor (FL-3) cats since the control animal was also capable of four-limbed locomotion, but was stimulated only briefly (∼1 min), 8 h before perfusion.

#### DβH/*Fos* Immunohistochemistry

**FIGURE 6:**
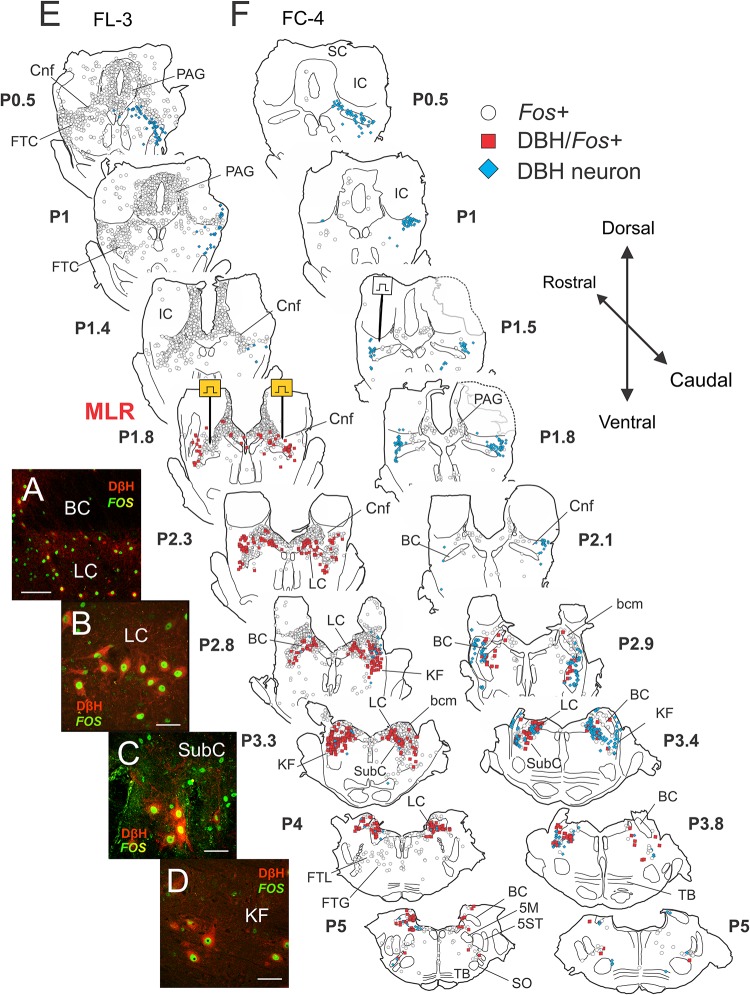
Noradrenergic neurons within the locus ceruleus (LC), subceruleus (SubC), and Kölliker–Fuse (KF) nuclei show activity-dependent *Fos* labeling following MLR-evoked fictive locomotion in the decerebrate cat. **(A–D)** Immunocytochemical identification of noradrenergic locomotor-activated neurons co-stained with *Fos* (*green*) and dopamine-β-hydroxylase or DβH (red). Confocal photomicrographs of cells located in LC **(A,B)**, SubC **(C)**, and KF **(D)** of fictive locomotor cat 3 (FL-3). Note that some cells showed only *Fos* labeling. **(E,F)** Camera lucida drawings showing distribution of *Fos*, DβH/*Fos*, and DβH immunoreactive neurons in locomotor and control cats, respectively. MLR stimulation electrodes were located at posterior 1.8 (P1.8; Berman, 1968). Testing electrode in control cat FC-4 was found at P1.5. In the MLR locomotor cat, abundant *Fos*-labeled neurons were observed in the CnF (site of stimulation), the bcm or SubCnF region, the PAG, LDT, LC, and the FTL. Large numbers of *Fos*+ neurons in the LC/SubC and KF stained positive for DβH. A few scattered *Fos*+ cells were observed in the inferior colliculus (IC) and superior colliculus (SC). The control animal was only briefly stimulated (50 s of locomotor activity total), indicating comparable health of the brain stem and spinal cord. Fewer *Fos*-labeled neurons were observed in FC-4, although there were slightly more *Fos*-labeled cells on the side of the MLR stimulation test. Scale bar: 100 μm **(A)** and 40 μm **(B–D)**.

Dopamine-beta-hydroxylase IHC was used to quantify the number of noradrenergic neurons in midbrain and pons activated during MLR-evoked lcocomotion. Numerous noradrenergic neurons were observed in the LC, SubC, and KF of both locomotor and control animals. Noradrenergic neurons were medium-sized oval, fusiform, or round (Figures 6A–D). Maps of *Fos-* and/or DBH-stained neurons within the brainstems of locomotor and control cats are illustrated in Figures 6E,F.

##### Locomotor experiment

As observed in Study 1 animals, abundant *Fos*-labeled neurons were observed in the CnF (sites of stimulation), the bcm or SubCnF region, FTC, PAG, LDT, LC, SubC, and KF of cat FL-3. *Fos*+ cells were generally symmetrically distributed in this bilaterally stimulated animal. Fewer neurons were labeled in the superior (SC) and IC pontine FTL and FTG (Figure 6E – P4 and P5). Large numbers of *Fos*+ neurons in the LC/SubC and KF stained positive for DβH (Figures 6A–D), indicating that many of the Fos+ labeled cells in the LC region in Study 1 were likely noradrenergic (Figure 5C). While the majority of *Fos*+ noradrenergic neurons were located 1–2 mm away from the sites of stimulation, a scattering of *Fos*+ noradrenergic neurons were also observed in the parabrachial region nearer to the electrode stimulation site, as reported previously (Steeves et al., 1976).

##### Non-locomotor control

Like Study 1, few *Fos*-labeled cells were observed in the control animal (Figure 6F) than in the locomotor animal (Figure 6E). Furthermore, relatively few noradrenergic neurons in the control animal showed *Fos* expression. Interestingly, *Fos* co-expression in some sections was slightly higher on the side of the stimulation used to demonstrate that the control animal was capable of MLR-evoked locomotion.

##### Distribution of *Fos*-labeled DβH neurons

**FIGURE 7:**
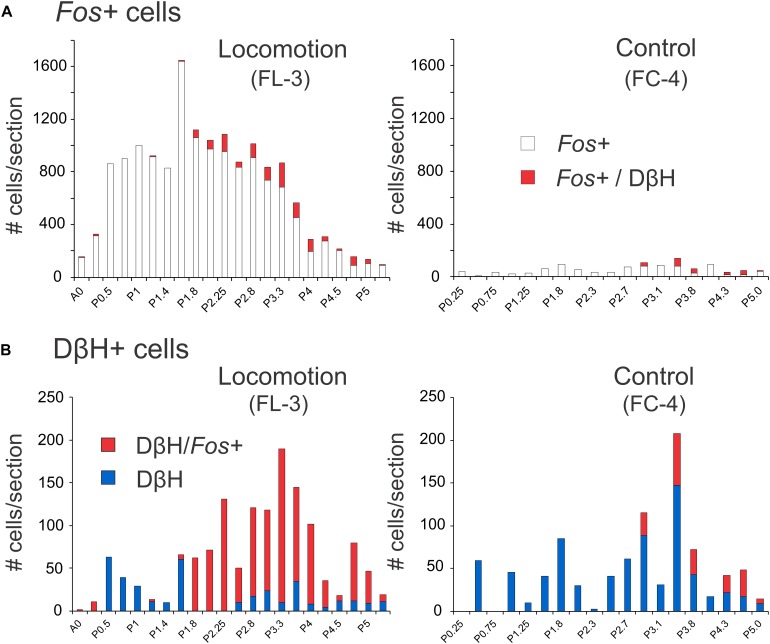
Distribution of *Fos*+ neurons with or without co-labeling with DβH in the midbrain and pons following bilateral stimulation of the MLR. **(A)** The number of *Fos*-labeled cells increased tremendously in FL-3 compared to the non-locomotor control animal (FC-4). **(B)** Noradrenergic neurons showed dramatic increases in *Fos* expression in the locomotor animal. Numeration includes all labeled cells from single sections at the indicated levels.

The rostro-caudal distribution of *Fos*+ cells observed in the brainstem of locomotor (FL-3) and control (FC-4) cats is illustrated in Figure 7A. In FL-3, the greatest number of *Fos*+ cells was found between P0.5 and P3.3, with a peak at P1.6 near the MLR stimulation site located at P1.8 (note that the electrolytic lesion used to mark the stimulation sites likely resulted in an underestimate of the number of *Fos*+ cells at that level). This represented a 6–144-fold increase in the number of *Fos*+ cells compared to the control animal. At more caudal levels (P3.7–5.0), the number of *Fos*-labeled neurons in the locomotor animal was 3–10 times greater than the control. The number of *Fos* immunoreactive noradrenergic neurons was also dramatically increased in the locomotor animal compared to the control (Figure 7B). The largest number of *Fos*+/DβH cells in FL-3 was found between P2.25 and P4.0 and peaked at P3.3 where all three noradrenergic nuclei (LC, SubC, and KF) were present. Between P2 and P4, ∼75–100% of noradrenergic cells showed *Fos* co-expression. In contrast, relatively few noradrenergic neurons between P2-4 showed *Fos* labeling in the control animal (0–37%).

#### ChAT/*Fos* Immunohistochemistry

**FIGURE 8:**
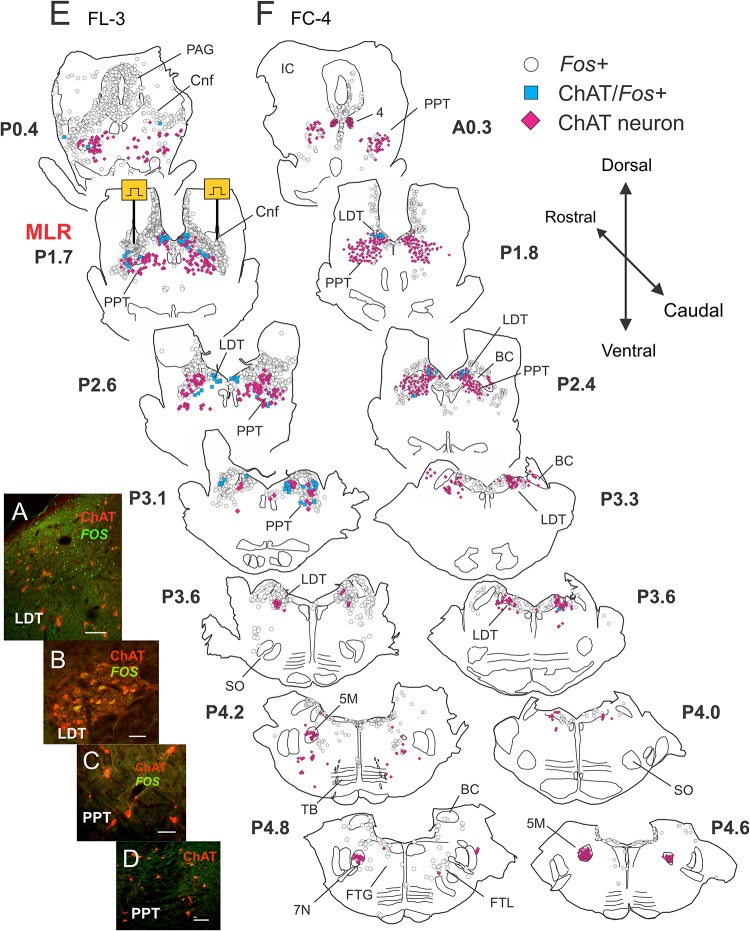
Few cholinergic cells in midbrain and pons show activity dependent *Fos* labeling following MLR-evoked fictive locomotion in the decerebrate cat. **(A–D)** Confocal photomicrographs demonstrating *Fos* (*green*) and/or choline acetyltransferase (ChAT, *red*) immunoreactive cells in the brainstem between levels P0-5. Micrographs taken of cells located in the LDT **(A,B)** or PPT **(C,D)** of fictive locomotor cat FL-3. Few ChAT neurons in the PPT showed *Fos* labeling. **(E,F)** Camera lucida drawings showing distribution of *Fos*, ChAT/*Fos*, and ChAT immunoreactive neurons in locomotor FL-3 and control FC-4 cats, respectively. MLR stimulation sites, marked electrolytically, were located at approximately P1.7 (Berman, 1968) in FL-3. As in Figure 4, the MLR locomotor cat show abundant *Fos*-labeled neurons were observed in the CnF, bcm, PAG, LDT, LC, SubC, and the FTL. Some cholinergic neurons in the PPT and LDT (about equal numbers) showed *Fos* labeling although many non-cholinergic neurons in these areas also showed *Fos* expression. Relatively few *Fos*+ neurons were observed in the control animal. Scale bar: 100 μm **(A,D)** and 50 μm **(B,C)**.

Choline acetyltransferase IHC was used to quantify the number of cholinergic neurons in midbrain and pons activated during MLR-evoked locomotion. Numerous cholinergic neurons were observed in the PPT, LDT, trochlear (4), and motor trigeminal (5M) nuclei of both locomotor and control animals. The neurons were medium-sized, irregular multipolar shaped. Photomicrographs taken from single sections through the LDT and PPT of cat FL-3 are illustrated in Figures 8A–D. Maps of *Fos-* and/or ChAT-stained neurons within the brainstems of locomotor and control cats are illustrated in Figures 8E,F. At their most rostral location, PPT neurons were observed more ventrolaterally within the tegmentum. Caudally, PPT neurons were observed loosely scattered around or ventral to the BC in areas occupied by noradrenergic neurons stained in other adjacent sections (Figures 6E,F).

##### Locomotor experiment

As observed in sections stained for *Fos* and DβH (Figures 6E,F), a large number of *Fos*-labeled neurons were observed in the CnF (sites of stimulation), bcm, PPT, FTC, PAG, LDT, LC, SubC, and KF. Caudally, a few neurons were labeled in the FTL and FTG (Figure 8E – P5.5 and P6). Relatively few cholinergic PPT neurons, however, stained positive for *Fos* (Figure 8E). Cholinergic PPT neurons were located ventral to sites producing the best locomotor response to electrical stimulation. In the LDT, a number of cholinergic neurons also stained positive for *Fos*. This group of cells accounted for about 50% of the double-labeled cholinergic neurons in the locomotor animal. Cholinergic motoneurons in cranial nerve nuclei [trochlear nucleus (4) and motor trigeminal nucleus (5M)] did not stain for *Fos*.

##### Non-locomotor control

As before, fewer *Fos*-labeled cells were observed in the control animal (Figure 8F) than in the fictive locomotor animal (Figure 8E). Likewise, few PPT cholinergic neurons showed *Fos* co-expression although there were a number of double labeled cells in the LDT. As in locomotor animals, no motor nuclei (4 and 5M) were double labeled.

##### Distribution of *Fos*-labeled ChAT neurons

**FIGURE 9:**
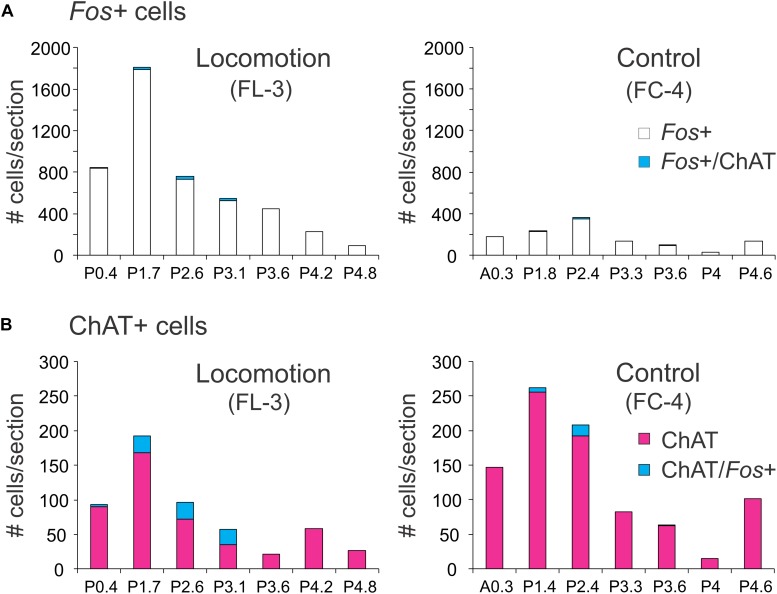
Distribution of *Fos*+ neurons with or without co-labeling with ChAT in the midbrain and pons following bilateral stimulation of the MLR. **(A)** The number of *Fos*-labeled cells increased tremendously compared to the non-locomotor control animal. Relatively few *Fos*+ neurons were cholinergic. **(B)** Comparison of cholinergic neurons with or without *Fos* expression in the locomotor and control animals. Numeration includes all labeled cells from single sections at the indicated levels.

The rostro-caudal distribution of ChAT and/or *Fos*+ cells in control and locomotor animals is shown in Figure 9. In FL-3, *Fos*+ cells peaked at P1.7 near the MLR stimulation sites (lesion visible at this level). This represented an approximately eightfold increase compared to the control. At more caudal levels (∼P2.4–4.2), the number of *Fos*-labeled neurons showed a two to sevenfold increase in the locomotor cat. The number of *Fos*-immunoreactive cholinergic neurons was slightly increased in the locomotor animal (Figure 9B), the increase split between cholinergic neurons in the PPT and LDT. This increase was much less than that observed for noradrenergic neurons within the same area (Figure 7B).

#### 5-HT/*Fos* Immunohistochemistry

**FIGURE 10:**
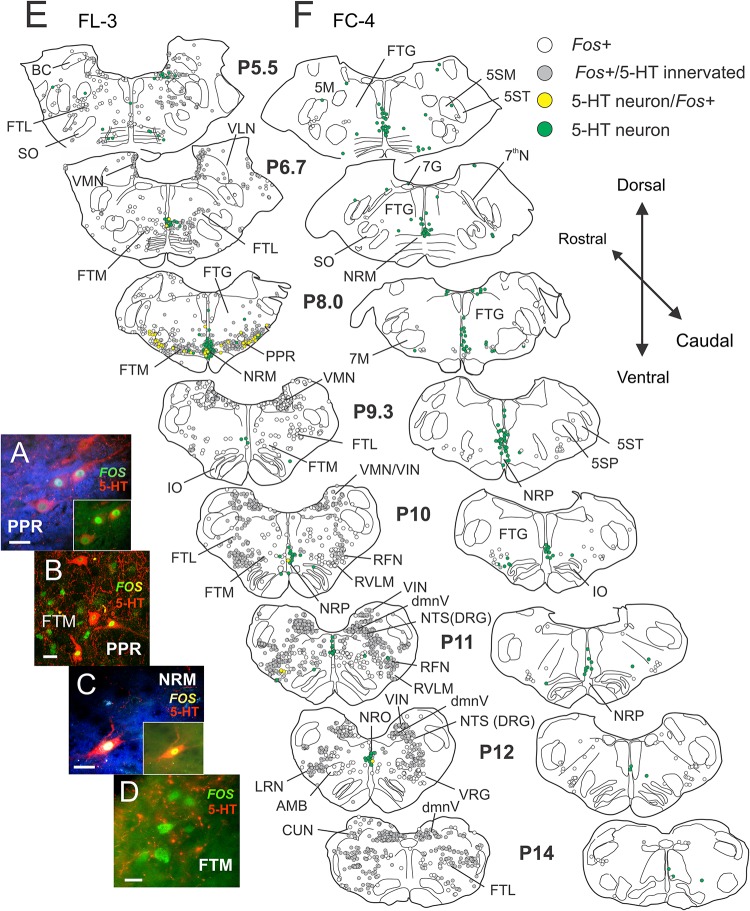
Locomotor activated pontomedullary neurons: serotonergic cells within the raphe and parapyramidal region show activity-dependent *Fos* labeling following MLR-evoked fictive locomotion in the decerebrate cat. **(A–D)** Confocal photomicrographs of locomotor-activated neurons from FL-3 showing *Fos* nuclear labeling (*green*) with and without cytoplasmic co-localization of 5-HT (*red*). Photomicrographs taken from FTM, PPR, and nucleus raphe magnus (NRM). Micrographs enhanced (blue background) in **(A)** and **(C)** to better illustrate fine serotonergic fibers and varicosities in surrounding neuropil (insets show original micrographs). Note that *Fos*+ cells in the FTM (and other regions of the reticular formation) are surrounded by a dense network of serotonergic fibers, likely making close contacts with the neurons. **(E,F)** Camera lucida drawings of single sections between posterior levels P5.5–14 showing distribution of *Fos*, 5-HT/*Fos*+, and 5-HT immunoreactive neurons in locomotor FL-3 and control FC-4 cats, respectively. *Fos* neurons with serotonergic contacts (gray) are indicated. In the MLR locomotor cat, abundant *Fos*-labeled neurons were observed in the FTM, FTL, PPR, NRM, medial vestibular nucleus (VMN), inferior vestibular nucleus (VIN), dorsal motor nucleus of the vagus (dmnV), rostral ventrolateral medulla (RVLM) and the nucleus tractus solitarii (NTS), and retrofacial nucleus (RFN) of the dorsal respiratory and ventral respiratory groups (DRG and VRG), respectively. Many locomotor-activated neurons in the PPR and NRM were positive for 5-HT. Few *Fos*+ neurons were observed in the control animal. Scale bar: 20 μm **(A–C)**; 10 μm **(D)**.

Numerous serotonergic neurons were observed in the NRM, pallidus (NRP), obscurus (NRO), and PPR of locomotor and control animals. Photomicrographs of serotonergic and non-serotonergic reticular (FTM) neurons taken from single sections of the brainstem of cat FL-3 are illustrated in Figures 10A–D. Serotonergic neurons were medium-sized, oval, or fusiform and in the PPR, were intermingled with other non-serotonergic neurons. In all areas of the medulla, serotonergic fibers formed a dense network surrounding many *Fos*-stained neurons (Figure 10D) and many cells in reticular and other areas appeared to be innervated by them (Noga et al., 2009). Maps of *Fos* and/or 5-HT stained neurons within the brainstems of locomotor and control cats are illustrated in Figures 10E,F.

##### Locomotor experiment

Many *Fos*-labeled cells were observed in the pons and medulla (P5.5–P14) following the locomotor task (Figure 10E). The distribution of these neurons was mostly symmetrical in this bilaterally stimulated animal. Abundant *Fos*-labeled neurons were found in the FTM, dorsal to the TB, and pyramids in a region laterally bounded by the superior olive (SO) and facial motor nucleus (7M). Relatively fewer cells were labeled in the FTG and more dorsally located FTL in this rostral area. This pattern of activated neurons corresponded with the MLR termination pattern described by Steeves and Jordan (1984). At the P8.5 level and caudally, ventrally located *Fos*+ neurons were found extending more laterally in the FTL. Numerous *Fos*+ neurons were also observed in the VMN and the VIN in rostral and caudal medulla. Relatively few labeled cells were observed within the lateral VLN. In caudal areas of the medulla, at the level of the inferior olivary nuclei (IO), large numbers of *Fos*+ cells were observed in the NTS of the dorsal respiratory group (DRG), the RFN of the VRG, the LRN, the RVLM, and the dmnV. Moderate labeling was observed in the FTM in caudal areas and scattered labeling of neurons were seen in the FTG. A small number of *Fos*+ cells was found within or bordering the AMB. Several serotonergic neurons in NRM were double labeled with *Fos*. Laterally, a large number of the serotonergic neurons within the PPR were also positive for *Fos* (P8.5).

##### Non-locomotor control

Like that observed in Study 1, maps constructed from brainstem segments in the control (FC-4) animal showed relatively few *Fos*-labeled neurons (Figure 10F). The difference between the control (Figure 10F) and fictive locomotor animals (Figure 10E) was striking. No serotonergic neurons in the control animal showed *Fos* labeling.

##### Distribution of *Fos*-labeled 5-HT neurons

**FIGURE 11:**
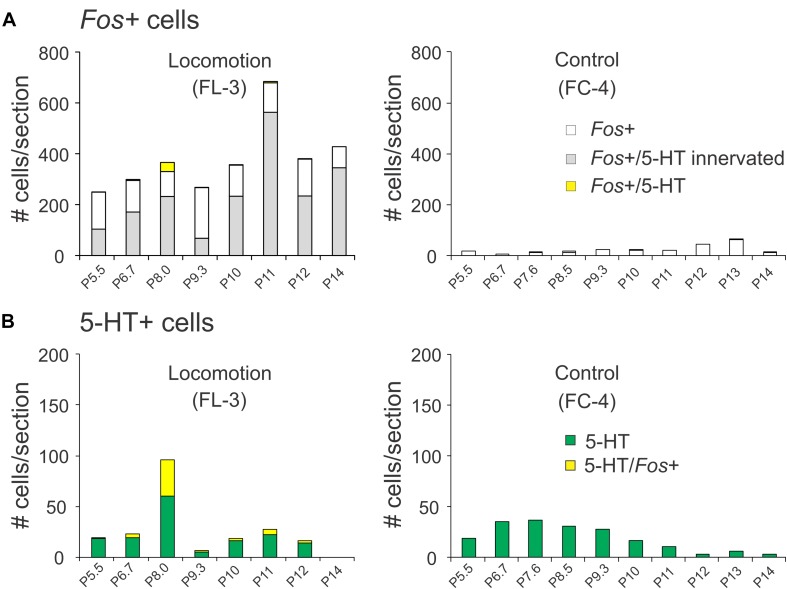
Distribution of *Fos*+ neurons with or without co-labeling with 5-HT in the pons/medulla following bilateral stimulation of the MLR. **(A)** The number of *Fos*-labeled cells increased tremendously compared to the non-locomotor control animal. A small number of *Fos*+ neurons were serotonergic. **(B)** Comparison of serotonergic neurons with or without *Fos* expression in the locomotor and control animals. Most serotonergic neurons within the PPR were positive for *Fos*. Numeration includes all labeled cells from single sections at the indicated levels.

The rostro-caudal distribution of 5-HT and/or *Fos*+ cells for control and locomotion experiments is shown in Figure 11. In the locomotor animal, the number of *Fos*+ cells/section found between P5.5 and P14 ranged from 250 to 684 and peaked at the P11 level in the caudal medulla (Figure 11A). In contrast, the number of *Fos*+ neurons/section in the control animal ranged between 7 and 64 neurons. At the peak level, this represented an approximately 34-fold increase in the number of *Fos*+ neurons in the locomotor animal compared to control. At other levels this increase ranged from 8- to 43-fold. Serotonergic immunoreactive boutons were found in close contact with many of these *Fos*+ neurons (Figure 11A). Overall, the percentage of *Fos*+ cells contacted by serotonergic fibers was ∼60% (Figure 7B) with a range between 25 and 83%. The number of *Fos*-immunoreactive serotonergic neurons was increased in the locomotor animal compared to control (Figure 11B). The largest number of *Fos*+/5-HT cells was found at P8.5 at the level of the NRM and PPR. At this level, 38.5% of serotonergic neurons expressed *Fos* protein. In contrast, no serotonergic neurons between P5.5 and P14 in the control animal expressed *Fos*.

##### Overview of labeled cells in locomotor and control animals – Study 2

**FIGURE 12:**
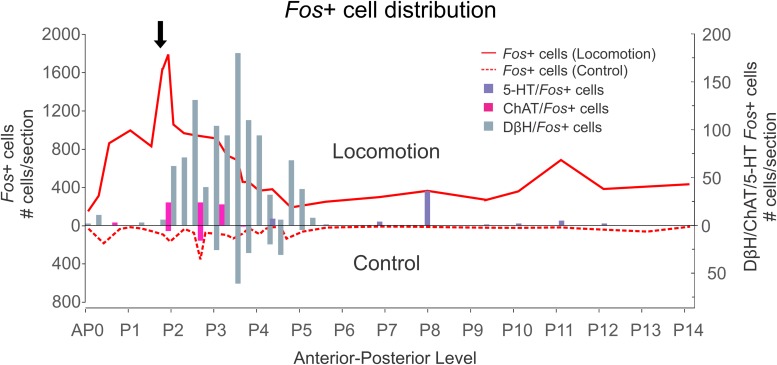
*Fos*+ cell distribution within brainstem following MLR-evoked fictive locomotion. Numeration includes all labeled cells from single sections at the indicated AP levels. The numbers of activated neurons in locomotor and control animals are indicated above and below the *X*-axis, respectively. Scale bar for noradrenergic, cholinergic, and serotonergic neurons indicated on right side of diagram. MLR stimulation level is indicated by *arrow*. Data from FL-3 and FC-4.

The distribution of brainstem *Fos*+ neurons in control and animals subject to MLR-evoked fictive locomotion is plotted in Figure 12.

## Discussion

### General Observations and Limitations of the Study

In the present experiments we have documented the location of brainstem neurons activated during MLR-evoked locomotion in the precollicular–postmammillary decerebrate cat and examined their correspondence to serotonergic, noradrenergic, and cholinergic phenotypes. Fictive locomotion experiments were conducted to determine the activation pattern produced from centrally driven locomotor pathways. Due to the nature of these experiments, most of the data presented in this study is on individual animals, with some minor differences seen between animals; however, the differences demonstrated between MLR-stimulated and control animals was strongly significant. The results provide evidence in support of the idea that the anatomical equivalent of the MLR is the CnF and/or SubCnF region rather than the cholinergic PPT (Jordan, 1998; Takakusaki et al., 2016). Furthermore, it shows that MLR stimulation activates both reticular and monoaminergic neurons in parallel, providing anatomical and functional validation for centrally mediated monoaminergic neuromodulation of spinal locomotor circuitry during evoked locomotion (Noga et al., 2009, 2011, 2017). Lastly, the results also show that MLR stimulation activates neurons within vestibular, cardiovascular, and respiratory areas. Overall, these results demonstrate a complex neuromodulation pattern of brainstem neurons that integrate the kinematic, dynamic, and metabolic facets of locomotor activity induced by electrical stimulation.

### Mesencephalic Locomotor Region

Historically, two adjacent nuclei, the CnF and the PPT, have been proposed as putative structural correlates of the MLR and two schools of thought have emerged in support of one or the other nucleus. Much of the preclinical literature, including Shik et al. (1966) original description, has supported the more dorsally located CnF, where electrical mapping studies consistently show it to promote locomotion (Takakusaki et al., 2003). Others have favored the more ventral, cholinergic cell-containing PPT (Garcia-Rill et al., 1986, 1987, 2011), despite its more varied electrical mapping results (Takakusaki et al., 2003, 2016). Data from the present study are discussed below with respect to this and other recent studies of this area of the midbrain.

#### Cuneiform Nucleus and the Sub-Cuneiform Region

The lowest electrical threshold sites for initiation of locomotion in the present study were found within the boundaries of the CnF nucleus and SubCnF region, thereby defining the MLR (see also Takakusaki, 2008, 2013; Takakusaki et al., 2016). Extensive labeling of neurons in these sites was observed, the majority of which are likely glutamatergic (Mena-Segovia et al., 2009; Wang and Morales, 2009). Cells in these areas are consistently labeled in studies examining locomotor activated neurons with *Fos* IHC (e.g., Silveira et al., 1995; Brudzynski and Wang, 1996; Iwamoto et al., 1996; Lamprea et al., 2002; Ferreira-Netto et al., 2005). Recent optogenetic studies show that glutamatergic CnF neurons are capable of initiating locomotion at short latencies, through a range of gait patterns and speeds (Roseberry et al., 2016; Capelli et al., 2017; Caggiano et al., 2018; Josset et al., 2018). Importantly, it is glutamatergic mesencephalic reticular formation neurons, including regions of the CnF, SubCnF, and PPT, that are activated during treadmill locomotion and which may code for locomotor speed (Roseberry et al., 2016; Caggiano et al., 2018). Cholinergic neurons, in contrast are characterized by repetitive, slow firing (Takakusaki et al., 1996). In non-human primates, rhythmically active cells are preferentially located in more dorsal CnF and SubCnF locations than tonically activated ones (Goetz et al., 2016). These latter cells are located within a region with higher densities of choline acetyl transferase labeled (cholinergic) neurons, corresponding to the PPT. GABAergic CnF neurons (Mena-Segovia et al., 2009; Wang and Morales, 2009) cannot initiate locomotion and rather, block locomotion when activated (Roseberry et al., 2016). If they were activated by electrical stimulation in the present study, their influence was overcome by activation of other neurons.

#### Pedunculopontine Tegmental Nucleus and Other Cholinergic Nuclei

The classically defined cholinergic PPT nucleus has long been considered a component of the MLR (Garcia-Rill et al., 1986, 1987, 2011) but the role of cholinergic PPT neurons in locomotion is controversial. In the present study, we examined neuronal *Fos* expression in the area encompassing the PPT and the adjacent cholinergic nucleus, LDT. Cholinergic neurons of the PPT and LDT were distributed within the mesopontine tegmentum as described previously (Jones and Beaudet, 1987). These neurons were not co-extensive with the low threshold locomotor producing sites within the CnF and SubCnF. Based on the known anatomical projections of the CnF and SubCnF (Steeves and Jordan, 1984; Caggiano et al., 2018) and on anticipated but limited current spread, stimulation of these low threshold MLR sites would be expected to activate some cells within this area of the tegmentum. However, relatively few cholinergic PPT/LDT neurons were activated compared to non-cholinergic *Fos*+ neurons (Figures 8E, 9). Overall, this data support the growing body of evidence that cholinergic neuron activation does not play a principal role in MLR-evoked locomotion. This is consistent with our study showing that cholinergic antagonists fail to block MLR-evoked locomotion in decerebrate cats (Jordan et al., 2014) and data from Takakusaki et al. (2016) showing that electrical stimulation of PPT results in a muscarinic-sensitive motor inhibition. Selective deletion of the vesicular acetylcholine transporter also does not abolish open field locomotion nor affect locomotor coordination but may result in hyperactivity and balance problems in mature animals (Janickova et al., 2017). The results are also consistent with recent studies in rodents which show that optogenetic stimulation of cholinergic PPT neurons does not elicit locomotion in stationary animals (Roseberry et al., 2016; Caggiano et al., 2018; Josset et al., 2018). Cholinergic neurons may, however, modulate ongoing locomotion, producing accelerating (Roseberry et al., 2016) or decelerating effects on locomotor speed (Caggiano et al., 2018; Josset et al., 2018). This modulation is unlikely to result from co-release of glutamate or GABA (Roseberry et al., 2016; see also Wang and Morales, 2009) and could be the result of cholinergic action on neurons of the substantia nigra pars compacta (SNc) and VTA (see the section “Ascending Pathways”) (Dautan et al., 2016; Xiao et al., 2016), CnF (Jin et al., 2016), and/or reticular formation (Tebēcis, 1973; Shiromani et al., 1990; Smetana et al., 2010). Within the LDT, a small, but similar number of cholinergic neurons also stained positive for *Fos* (Figures 8A,B,E). The LDT is thought to play a role in arousal, eye movements, learning and reward, visual orienting, and sensory-motor patterns, possibly via projections to the VTA and SNc (Wang and Morales, 2009; for review, see Martinez-Gonzalez et al., 2011).

Non-cholinergic neurons within the PPT, LDT, and adjacent area (Jones and Beaudet, 1987; Usunoff et al., 2003; Wang and Morales, 2009) were also activated by electrical stimulation of the MLR. Photo-activation of glutamatergic PPT neurons is reported to induce low-speed locomotion from rest in a subset of trials (∼50%), but with long onset latency and requiring high frequency (50 Hz) stimulation (Caggiano et al., 2018). This has led to the suggestion that glutamatergic PPT neurons may be involved in explorative locomotor behavior (Caggiano et al., 2018). In support of this suggestion, these authors have shown that both the CnF and the PPT glutamatergic neurons project predominantly ipsilaterally, to locomotor areas of the MedRF (Noga et al., 2003). In a different study, however, glutamatergic PPT cell activation not only failed to initiate locomotion, it also decelerated and stopped ongoing locomotion (Josset et al., 2018; see also Takakusaki et al., 2016). Partial or complete lesions of the PPT (affecting all neuronal types) also fail to result in gait deficits (Gut and Winn, 2015) indicating that such modulatory effects on locomotion are likely compensated for by other modulatory systems. Further careful electrophysiological studies are needed to establish the role for the PPT.

Although more concentrated within the rostral pole of the PPT (Pienaar et al., 2017), GABAergic PPT neurons cannot initiate locomotion and rather, block locomotion when activated (Roseberry et al., 2016; Caggiano et al., 2018). If they were activated by electrical stimulation in the present study, their influence was minimal.

### Ascending Pathways

*Fos* expression was elevated in the ipsilateral PAG (Figure 4A), an important mediator of defensive behavior including escape locomotion (Koutsikou et al., 2017). More ventrally, labeling was observed within the FTC, FF, and VTA in a sagittaly continuous band of activated neurons. Bilateral MLR stimulation produced symmetrical *Fos* expression (Figures 6E, 8E) within the PAG and FTC, and may be the activation pattern for rectilinear locomotion with balanced bilateral MLR activity (Noga and Opris, 2017b; see the section “Asymmetry in Brainstem Circuits”). This functional connectivity is consistent with anterograde tracer studies targeting the MLR and/or the CnF nucleus (Edwards, 1975; Edwards and de Olmos, 1976; Steeves and Jordan, 1984; Sotnichenko, 1985). The strong interconnection of the CnF, PAG (Edwards and de Olmos, 1976; Mantyh, 1983; Steeves and Jordan, 1984; Sotnichenko, 1985; Ferreira-Netto et al., 2005; Dampney et al., 2013; Caggiano et al., 2018), and the limbic system (see Koutsikou et al., 2017) indicates that the MLR plays an important role in the integration of complex motor behaviors related to defensive behavior (Sinnamon, 1993; Jordan, 1998).

Neurons within the VTA showed increased *Fos* activity (Figure 4A). The VTA contains dopaminergic neurons involved in goal-directed behavior and reinforcement-learning (Wise, 2004). It receives a direct input from non-catecholaminergic neurons of the PAG (Suckow et al., 2013) and from cholinergic and glutamatergic neurons of the PPT and LDT (Mena-Segovia and Bolam, 2017). Stimulation of cholinergic PPT terminals within the VTA activates dopaminergic neurons and transiently increases locomotor activity (Dautan et al., 2016). In contrast, LDT cholinergic neuron activation decreases locomotion (Dautan et al., 2016) and results in reward reinforcement (Xiao et al., 2016). These differential effects are likely due to actions on different neurons within the VTA. PPT glutamatergic neurons also increase arousal and drive motivated behavior via ascending projections, in part to the VTA (Kroeger et al., 2017; Yoo et al., 2017).

### Descending Pathways

#### Reticular Formation

The major output pathway of the brainstem for activation of locomotor circuits is the RS pathway originating in the rostral medulla (Orlovskii, 1970; Garcia-Rill et al., 1983; Shefchyk et al., 1984; Steeves and Jordan, 1984; Garcia-Rill and Skinner, 1987; Noga et al., 1988, 1991, 2003). In the present study, neurons in the nucleus reticularis magnocellularis (FTM) were the primary reticular neurons activated in this region, dorsal to the TB and pyramids. Relatively few neurons were labeled in the FTG, although more posteriorly we observed labeling within the FTL (Figure 10; P9.3) which gradually merged with areas corresponding to the cardiorespiratory regions of the caudal medulla (see the section “Coupling of Neuronal Networks”). An asymmetrical activation pattern was observed with unilateral stimulation of the MLR (Figures 3–5), mirroring the anatomical projection pattern of the MLR (Steeves and Jordan, 1984). The implications of this pattern are discussed below (see the section “Asymmetry in Brainstem Circuits”).

One candidate RS neuron mediating MLR-evoked locomotion is the Lhx3/Chx10-expressing neuron in the mouse (Bretzner and Brownstone, 2013). These neurons are glutamatergic, are targets of MLR (CnF) projections, support tonic repetitive firing, project to the spinal cord, and are activated (express *Fos*) during wheel running or treadmill locomotion. They are found in the ventral and α (FTM in the cat) parts of the gigantocellular reticular nuclei (together termed α/vGRN or GiA/GiV). Optogenetic studies in mice have shown that activation of glutamatergic neurons within the LPGi, a caudal subgroup of the magnocellular nucleus, can also trigger continuous locomotion (Capelli et al., 2017). Neurons in this area harbor terminals of MLR (CnF/PPT) efferents and express *Fos* after locomotion (Capelli et al., 2017). Some neurons in this lateral subdivision of the FTM caudal to 7M were also labeled in the present study. However, MLR (CnF) projections in the cat do not extend much more caudally than 7M (Steeves and Jordan, 1984), indicating that activation of reticular neurons at more caudal levels may be indirect, via local circuits within the brainstem (Shimamura et al., 1980). Further studies are needed to clarify whether there are species differences that may account for these discrepancies (see also Caggiano et al., 2018).

Interestingly, a large percentage of the MedRF neurons were innervated by serotonergic fibers. Such innervation of variously sized cells in the reticular formation has been described before (Kobayashi et al., 1994; see also Gao and Mason, 1997; Viana Di Prisco) as well as for vestibular (Halberstadt and Balaban, 2003) and cardio-respiratory neurons (see the section “Coupling of Neuronal Networks”). The serotonergic innervation of RS neurons may thus provide the basis for a neuromodulatory influence of 5-HT on brainstem circuits (Takakusaki et al., 1993), in addition to its effects on spinal circuits for locomotion (see Schmidt and Jordan, 2000).

#### Monoaminergic Neurons

As discussed in the section “Introduction,” monoamines play a key role in the activation of spinal locomotor networks. The present study now confirms that monoaminergic neurons are activated during MLR-evoked locomotion (Figures 6, 10), with increased activity-dependent labeling of both catecholaminergic neurons of the LC, SubC, and KF nuclei and serotonergic neurons of the NRM and PPR. These nuclei are the primary source of the monoaminergic innervation of the spinal cord (Westlund et al., 1982; Clark and Proudfit, 1991a, b; Jones and Light, 1992). Monoaminergic neurons are likely activated by direct projections from the CnF and/or MLR (Edwards, 1975; Steeves and Jordan, 1984; Sotnichenko, 1985). Furthermore, both cerulear (Rasmussen et al., 1986) and raphe neurons are rhythmically active during overground locomotion (Veasey et al., 1995; Jacobs et al., 2002) and stimulation of the PPR in the neonatal rat also produces serotonergic receptor-dependent locomotor-like activity (Liu and Jordan, 2005). Taken together with our observation that stimulation of the MLR results in the spinal release of 5-HT and NE (Noga et al., 2017), these results provide the anatomical basis for the central control of locomotor activity by 5-HT and NE, in the absence of peripheral afferent feedback from moving limbs. Thus, in addition to RS command neurons (see the section “Reticular Formation”), monoaminergic neurons comprise a major component of the central descending pathways controlling locomotion.

### Coupling of Neuronal Networks

Cells in other brainstem nuclei show increased activity-dependent labeling following MLR-evoked locomotion. These include nuclei of cardiovascular, respiratory, and vestibular systems (Figure 10). Several studies have demonstrated that locomotor and respiratory rhythms are centrally coupled (DiMarco et al., 1983; Millhorn et al., 1987; Perségol et al., 1988; Kawahara et al., 1989, 1993; Ezure and Tanaka, 1997). Respiratory and cardiovascular networks are also coupled through peripheral feedback (Iwamoto et al., 1996) and/or central interconnections between the different pattern generators (Dick et al., 2009; Le Gal et al., 2014). Here we present functional and anatomical evidence for a central coupling of locomotor, respiratory, and cardiovascular networks (DiMarco et al., 1983; Eldgridge et al., 1985; Bell, 2006; Wienecke et al., 2015) as well as activation of neurons within the medial VLN following stimulation of the MLR. While most of the nuclei of the cardiovascular, respiratory, and vestibular systems in the cat are not directly innervated by the CnF/MLR (Edwards, 1975; Steeves and Jordan, 1984), other nuclei receiving projections from the MLR (e.g., monoaminergic system, see below) may act as intermediaries to modulate their activity.

#### Respiratory Nuclei

In the present study, stimulation of the MLR increased *Fos* expression in nuclei involved in respiratory control, including neurons in the NTS, RFN, and LRN of the dorsal and VRGs. Other nuclei that may modulate respiratory activity under specific conditions also showed increased *Fos* expression in the present study. These included neurons within the raphe/PPR region, LC, SubC, KF, PPT, and PAG. Although respiratory responses to MLR stimulation were not monitored in these animals, previous work has shown that stimulation of the hypothalamic and MLRs facilitate respiration (Eldgridge et al., 1981; DiMarco et al., 1983; Millhorn et al., 1987; Kawahara et al., 1989; Ezure and Tanaka, 1997). Serotonergic neurons from the raphe nuclei and parapyramidal region project to the dorsal (Voss et al., 1990) and ventral respiratory column and may also contribute to central chemoreception and respiratory control (Ribas-Salgueiro et al., 2005; DePuy et al., 2011; Morinaga et al., 2019). Evidence of their involvement in control of breathing in the present study comes from the observation that serotonin-immunoreactive boutons were found in close apposition to many of these neurons (see also Voss et al., 1990). LC and KF nuclei are also involved in the control of breathing (Dutschmann and Herbert, 2006; Gargaglioni et al., 2010; Dutschmann and Dick, 2012; Barnett et al., 2018). Effects are likely mediated by noradrenergic (Magalhães et al., 2018) or glutamatergic projections (in the case of the KF) (Herbert et al., 1990; Ezure and Tanaka, 2006; Yokota et al., 2007; Geerling et al., 2017). The major source of cholinergic innervation of the brainstem regions controlling breathing is from the PPT and LDT (Kubin and Fenik, 2004) and neurons from these nuclei may thus have contributed to the activation of medullary respiratory-related neurons in the present experiments (Chatonnet et al., 2003). Lastly, respiratory activity may be modulated by the PAG; indirectly through its projections to the CnF (from the dlPAG); or directly by projections to the parabrachial complex, midline medulla, and VRG (from the dmPAG, lPAG, and vlPAG; see Dampney et al., 2013).

#### Cardiovascular Nuclei

Stimulation of the MLR invariably increased blood pressure (Figure 2) and *Fos* expression in nuclei associated with cardiovascular regulation (Figure 10). Consistent with this observation, stimulation of the CnF in the anesthetized rat increases arterial blood pressure in the absence of locomotion. The effect on blood pressure may be mediated by activation of sympathoexcitatory neurons in the RVLM (Verberne, 1995), catecholaminergic neurons of the KF/parabrachial complex and LC (Lam et al., 1996; Shafei and Nasimi, 2011), the dorsal PAG (Lam et al., 1996), and/or serotonergic neurons of the caudal raphe nuclei (Lam and Verberne, 1997; DePuy et al., 2011). Neurons within the parapyramidal region also project to cardiovascular-related nuclei (NTS) and may increase mean arterial blood pressure, independent of the RVLM (Helke et al., 1989). Interestingly, cholinergic systems may counteract the pressor effect of CnF stimulation by acting directly on nuclei known to produce hypotension (Shafei et al., 2013). Lastly, a role of the dorsolateral (sympathoexcitatory) and ventrolateral (inhibitory) PAG in the regulation of cardiovascular function has also been demonstrated (e.g., Carrive and Bandler, 1991; Lovick, 1992; Subramanian and Holstege, 2014) possibly via the FTM, raphe nuclei (Gao et al., 1997; Hermann et al., 1997), RVLM, or CnF (see Dampney et al., 2013).

#### Vestibular Nuclei

Vestibular signals are important in the regulation of balance (Shinoda et al., 2006) and contribute to cardiovascular and respiratory regulation during movement (McCall et al., 2017). In the present study, *Fos* labeling was observed in the VMN and VIN, areas important for stabilization of the head during movement (Cullen, 2012). Relatively few neurons within the lateral VLN were labeled. Orientation and movement of the head in the walking cat are active processes but reflexes appear to play only a partial role in determining head movement during walking, indicating that signals from the centrally generated locomotor synergy must be the main drivers for head movements (Zubair et al., 2016). In contrast, although vestibulospinal neurons within the VLN are rhythmically active during locomotion (Orlovsky, 1972; Matsuyama and Drew, 2000), their rhythmic activity likely reflects hindlimb and labyrinthine inputs during walking (Arshian et al., 2014) rather than centrally generated activity. Supporting this, bilateral lesions of the VLN in decerebrate cats do not interrupt MLR-evoked locomotion (Jell et al., 1985). The VLN are not directly innervated by projections of the MLR (Steeves and Jordan, 1984). Possible sources of activation of these nuclei (reviewed by McCall et al., 2017) in the reduced paralyzed preparation may include cerebellar (fastigial) nuclei (Takakusaki et al., 2016) or spinal interneurons (Noga et al., 2009, 2011) signaling locomotor activity. Interestingly, afferent inputs that may contribute to the vestibulo-cardiovascular and respiratory reflex relayed through the VMN and VIN originate in the medullary and pontine reticular formation, LRN, and raphe nuclei (Jian et al., 2005) and it is possible that activity in these nuclei from centrally driven locomotor inputs (this study; Zubair et al., 2016) could, via the VLN, further enhance cardiovascular and respiratory center activation (Stocker et al., 1997) in addition to those nuclei described above.

### Asymmetry in Brainstem Circuits

As revealed in the present study, unilateral stimulation of the MLR produced an asymmetrical activation of brainstem neurons with *Fos* expression more commonly observed on the side of stimulation (Figures 3–5) even though bilateral locomotor activity was observed. This distribution reflects the anatomical projections of the MLR which are mostly uncrossed through the parabrachial region to the MedRF (Steeves and Jordan, 1984; see also Edwards, 1975; Sotnichenko, 1985). The results are consistent with the functional asymmetry of the RS output revealed by localized reversible cooling of the spinal cord in the decerebrate cat during fictive locomotion (Noga et al., 1995). Furthermore, they are consistent with electrophysiological studies which show that the majority of activated RS neurons project through the ventral funiculus on the same side as the stimulated MLR (Garcia-Rill and Skinner, 1987) to terminate on ipsilateral lumbar spinal neurons in the intermediate zone and ventral horn (Holstege and Kuypers, 1982; Capelli et al., 2017). Although fewer, projections from the MLR to the contralateral reticular formation (Steeves and Jordan, 1984; Capelli et al., 2017) and contralaterally/bilaterally projecting RS neurons (Peterson et al., 1975; Garcia-Rill and Skinner, 1987) likely account for the activation of RS neurons on the side opposite to stimulation [this study; Noga et al., 1995; see Humphries et al. (2006, 2007) for a discussion of intrinsic reticular network connections]. Finally, crossed spinal (Holstege and Kuypers, 1982; Kausz, 1991) or segmental pathways (Jankowska and Noga, 1990; Kjaerulff and Kiehn, 1997; Kremer and Lev-Tov, 1997; Matsuyama et al., 2004a) likely also contribute to the generation of bilateral locomotor activity with unilateral stimulation of the MLR. In such a way, secondary projection systems compensate for the anatomical asymmetry of the primary MLR projection. Thus, the spinal activation pattern produced by unilateral MLR stimulation is essentially symmetrical (Dai et al., 2005). While this experimental situation reveals the complex projections within brainstem and spinal cord, spontaneous locomotion likely would provide a more balanced descending output to the spinal locomotor centers (Noga and Opris, 2017b), reflective of the pattern of activation observed within the brainstem (Figures 6, 8, 10) and spinal cord (Noga et al., 2009, 2011) produced with bilateral MLR stimulation. In this situation, forward or rectilinear locomotion likely occurs through bilaterally symmetric commands transmitted by the MLR and RS pathways. In contrast, during turning movements, an asymmetric command may be generated and transmitted along RS pathways to modulate CPGs on one side. Such a command would need to overwhelm compensatory mechanisms from contralaterally projecting RS neurons and segmental commissural neurons (Noga et al., 2003; Matsuyama et al., 2004a). A theoretical model in mammals for symmetry breaking of rectilinear locomotion by adjusting the level of activity of components of the descending locomotor pathway has been presented (Noga and Opris, 2017b). In that model, steering of locomotor activity may be achieved by temporarily adjusting the balance of MLR and/or RS outputs – in essence, creating an asymmetrical drive on either side of the brainstem. Evidence in favor of such an organization at the RS level for steering of locomotor activity has recently been presented (Oueghlani et al., 2018).

### Descending Pathway for Initiation of Locomotion

**FIGURE 13:**
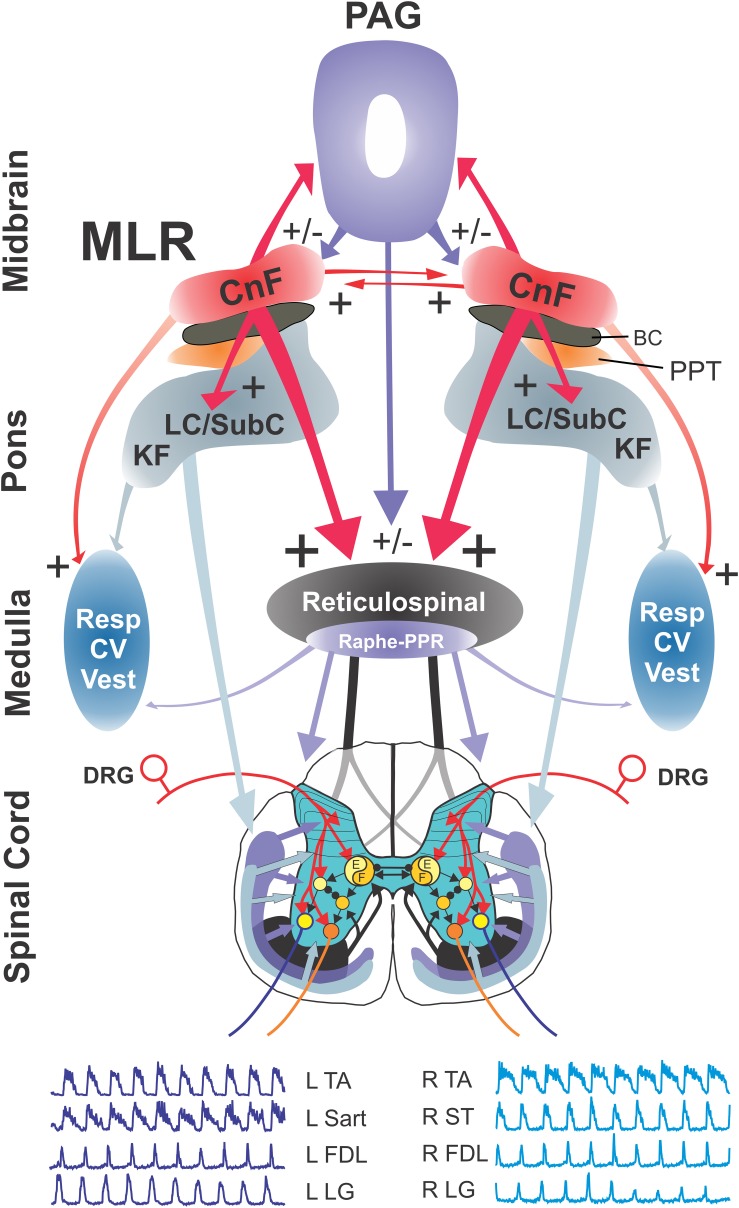
Model of brainstem pathways for initiation of locomotion in the cat. Relationships between the various components of the pathway activated by the MLR, the spinal central pattern generator for locomotion (CPG), and their output motoneurons for bilateral hindlimb locomotion. The model incorporates parallel activation of descending RS and neuromodulatory pathways originating in the catecholaminergic and serotonergic nuclei of the pons and medulla, in addition to the facilitation of cardiorespiratory and vestibular centers during locomotion. Glutamatergic neurons within the CnF and SubCnF region form the primary phenotype for initiation and control of locomotion (Caggiano et al., 2018; Josset et al., 2018). Glutamatergic PPT neurons may contribute to the initiation of low speed locomotion (Caggiano et al., 2018) although this is disputed (Josset et al., 2018). Cholinergic neurons do not initiate locomotion but may play a modulatory role for ongoing locomotion (Roseberry et al., 2016; Caggiano et al., 2018; Josset et al., 2018) possibly by their effects on other brainstem output neurons. RS neurons of the magnocellular reticular formation, which form the final common motor pathway of the brainstem, relay the central command for initiation of locomotion to the spinal locomotor central pattern generator ultimately activating hindlimb motoneurons (Shefchyk et al., 1984; Noga et al., 2003; Jordan et al., 2008). The model also incorporates known projections between CnF/MLR nuclei on each side of the midbrain (Edwards, 1975; Steeves and Jordan, 1984). At the spinal level, flexor (F) and extensor (E) components of the locomotor pattern generator are activated/modulated by descending bilateral RS and monoaminergic projections as well as by crossed excitatory (▶) and inhibitory (⚫) segmental projections from the generator opposite to it. Details of the rhythm and pattern components of the locomotor generator are omitted to emphasize general interconnections between them and their target neurons. Electroneurograms: FDL, flexor digitorum longus; LG, lateral gastrocnemius; Sart, sartorius; ST, semitendinosus; TA, tibialis anterior. R, right; L, left.

A new model of the descending pathway for the control of locomotion (after Noga et al., 2017) is presented in Figure 13, with the MLR representing a central node in the control of locomotion by higher brain centers. Data presented in the present study indicate that the anatomical locus of the MLR is the CnF/SubCnF region of the midbrain. Little evidence is found to support the participation of cholinergic neurons in the initiation of locomotion by electrical stimulation of this region, although a modulatory role of locomotor activity is possible (Roseberry et al., 2016; Caggiano et al., 2018; Josset et al., 2018). This is consistent with recent optogenetic studies that show that initiation of locomotor activity is primarily, if not exclusively, the result of activation of glutamatergic neurons within the CnF and SubCnf (Roseberry et al., 2016; Caggiano et al., 2018; Josset et al., 2018). The MLR is reciprocally connected with the contralateral MLR (Steeves and Jordan, 1984; Bayev et al., 1988) possibly facilitating/coordinating descending signal output on both sides of the brainstem, and with the PAG (Mantyh, 1983; Bayev et al., 1988; Sandner et al., 1992; Ferreira-Netto et al., 2005; Dampney et al., 2013; Caggiano et al., 2018) which may be important for the mediation of rapid defensive decision making or the mediation of locomotion during pursuit. Electrical stimulation of the MLR activates three primary brainstem targets affecting locomotor circuits within the spinal cord: RS, ceruleospinal and raphespinal. RS neurons located within the MedRF (FTM) comprise the primary “command pathway” for the initiation of locomotion (Shik et al., 1966, 1967; Orlovskii, 1970; Jordan, 1991; Noga et al., 2003). Glutamatergic RS neurons in this region activate spinal locomotor neurons (e.g., Douglas et al., 1993; Hägglund et al., 2010; Bretzner and Brownstone, 2013; Capelli et al., 2017). Activation of noradrenergic (LC, SubC, and KF) and serotonergic (NRM and PPR) neurons within the pons and medulla results in the rapid, widespread release of the neuromodulators NE and 5-HT in the spinal cord during locomotion (Noga et al., 2017). Central respiratory (Resp), cardiovascular (CV), and vestibular (Vest) neurons are also activated by MLR stimulation, either directly or indirectly (Voss et al., 1990; Nasimi et al., 2012; Damasceno et al., 2014), likely in anticipation of the increased metabolic and postural demands associated with locomotion. Additionally, MLR stimulation activates neurons within the PAG, an area important for mediating defensive behaviors (Deng et al., 2016).

The RS neurons of the MedRF (FTM) also have multiple inputs in addition to the MLR (Steeves and Jordan, 1984; Garcia-Rill and Skinner, 1987; Bretzner and Brownstone, 2013). They are innervated by the ipsilateral SLR (Sinnamon and Stopford, 1987; Takakusaki et al., 2016), the contralateral cerebellar locomotor region (Mori et al., 1998), the PAG (Mantyh, 1983; Dampney et al., 2013), the motor cortex via corticoreticular pathways (Matsuyama et al., 2004b), as well as various sensory systems (e.g., visual, auditory, and vestibular) (Furigo et al., 2010; Miller et al., 2017). Thus locomotion may be initiated by activation of the RF directly, bypassing the MLR (Shik et al., 1966; Noga et al., 1988; Mori et al., 1998; Bretzner and Brownstone, 2013; Capelli et al., 2017) or modulated by activation of sensory or neuromodulatory inputs to the RF (Antri et al., 2008; Smetana et al., 2010; Noga and Opris, 2017a, b; Oueghlani et al., 2018). The neuronal circuit selected for goal-directed locomotion may depend upon the behavioral context (Sinnamon, 1993), whether locomotion is required for either exploration, foraging, or defense (see Jordan, 1998; Takakusaki, 2008).

## Data Availability Statement

The datasets generated for this study are available on request to the corresponding author.

## Ethics Statement

The animal study was reviewed and approved by the University of Miami IACUC.

## Author Contributions

BN and LJ: conceptualization, supervision, and funding acquisition. BN, LJ, DJ, XD, and LV: methodology. BN, DJ, and XD: investigation. XD, IO, DJ, FS, LV, CL-H, and SX: formal analysis. BN, XD, IO, and LV: visualization. BN, XD, IO, and SC: writing – original draft. BN, LJ, IO, and SC: writing – review and editing.

## Conflict of Interest

The authors declare that the research was conducted in the absence of any commercial or financial relationships that could be construed as a potential conflict of interest.

## Abbreviations

4: trochlear nucleus
5M: motor trigeminal nucleus
5SL: laminar spinal trigeminal nucleus
5SP: spinal trigeminal nucleus
5ST: spinal trigeminal tract
7: facial nucleus
7G: genu of the facial nerve
7N: facial nerve
AMB: nucleus ambiguus
BC: brachium conjunctivum
bcm: marginal nucleus of the brachium conjunctivum
CI: inferior central nucleus
CnF: cuneiform nucleus
CU: cuneate nucleus
dmnV: dorsal motor nucleus of the vagus
DRG: dorsal root ganglion
FF: fields of Forel
FTC: central tegmental field
FTG: gigantocellular tegmental field
FTL: lateral tegmental field
FTM: magnocellular tegmental field
FTP: paralemniscal tegmental field
GR: gracile nucleus
IC: inferior colliculus
IO: inferior olive nucleus
KF: Kölliker–Fuse nucleus
LC: locus ceruleus
LDT: laterodorsal tegmental nucleus
LLD: dorsal nucleus of the lateral lemniscus
LRI: lateral reticular nucleus internal division
LRN: lateral reticular nucleus
MedRF: medial reticular formation
MLR: mesencephalic locomotor region
NRM: nucleus raphe magnus
NRO: nucleus raphe obscurus
NRP: nucleus raphe pallidus
NTS: nucleus tractus solitarii
P: pyramidal tract
PAG: periaqueductal gray
PPR: post-pyramidal nucleus of the raphe
PPT: pedunculopontine tegmental nucleus
RFN: retrofacial nucleus
RVLM: rostral ventrolateral medulla
SC: superior colliculus
SO: superior olivary nucleus
SubC: subceruleus
SubCnF: subcuneiform region
TB: trapezoid body
VIN: inferior vestibular nucleus
VLN: lateral vestibular nucleus
VMN: medial vestibular nucleus
VRG: ventral respiratory group
VTA: ventral tegmental area.
